# Comparison of efficacy and safety of various management options for large upper ureteric stones a systematic review and network meta-analysis

**DOI:** 10.1038/s41598-021-91364-3

**Published:** 2021-06-03

**Authors:** Gopal Sharma, Tarun Pareek, Shantanu Tyagi, Pawan Kaundal, Anuj Kumar Yadav, Yashasvi Thummala, Sudheer Kumar Devana

**Affiliations:** Department of Urology, Advanced Urology Center, Postgraduate Institute of Medical Education and Research (PGIMER), Level II, B Block, Sector 12, Chandigarh, 160012 India

**Keywords:** Biological techniques, Urology

## Abstract

To compare the safety and efficacy of various surgical modalities to manage large (> 1 cm) upper ureter stones. Systematic literature search was conducted to include all randomized studies comparing various treatment options for large (> 1 cm) upper ureteric stones. This review included 13 randomized studies with 1871 patients. Laparoscopic ureterolithotomy (LUL) and percutaneous nephrolithotomy (PNL) were superior to ureteroscopy (URS) and shockwave lithotripsy (SWL) for stone-free rates and need for auxiliary treatments. LUL and PNL were equally effective for stone-free rates and the need for auxiliary treatments. According to SUCRA values for stone-free rates and the need for auxiliary treatments, LUL was the best, followed by PNL. For the duration of surgery, there was no significant difference among all the techniques on network analyses, and SWL was the best according to SUCRA values. Length of hospital stay was significantly shorter for URS than LUL and PNL from network analysis, but there was no significant difference for the rest of the comparisons. Overall complications were similar in all the groups. According to the CINeMa approach, the confidence rating ranged from “very low” to “moderate” for various comparisons. LUL followed by PNL is the most efficacious treatment modality for upper ureteric stones compared to SWL and URS in terms of stone-free rates. However, due to the poor quality of included studies, further high-quality randomized studies are needed.

## Introduction

Management options for ureteric stones are manifold, ranging from conservative measures such as hydration, analgesics, and use of alfa blockers to various surgical options such as shock wave lithotripsy (SWL), ureterorenoscopy (URS), percutaneous nephrolithotomy (PNL) and laparoscopic ureterolithotomy (LUL). For ureteric stones less than 5 mm irrespective of the location of the stone, an initial trial of conservative therapy can be offered given the patient is mildly symptomatic and without complications like infection, deteriorating renal function or solitary kidney^1^. The latest European Association of Urology (EAU)^1^ guidelines endorse either SWL or URS as the first line management option for for upper ureteric stones less than 10 mm in size. Whereas for stones larger than 10 mm, URS is recommended as the first line treatment modality. Antegrade PNL is only used in specific situations such as the presence of large stone, impacted stone, simultaneous renal stones, failed URS or SWL and inability to obtain retrograde access (urinary diversions or ureteric strictures). LUL is another but more invasive surgical option for treating upper ureteric stones especially large and impacted stones. With multiple surgical options, there lies the controversy of choosing the best option for a given patient. There is no level I data to advocate one treatment over another; thus, selection of a particular treatment option depends on patient factors (fitness for anesthesia, acceptability to invasive or less invasive options or acceptability to low stone free rate), surgeon factors (skills and experience), stone factors (size, location, impaction) and anatomical factors (solitary kidney, unavailability of retrograde access). This network meta-analysis aimed to combine direct and indirect evidence comparing the different surgical options for upper ureteric stones in terms of safety and efficacy.

## Materials and methods

The primary objective of this study was to compare the efficacy and safety of various treatment options for upper ureteric stones of mean size > 1 cm. We performed this meta-analysis using the frequentist approach^2^, and standard guidelines were followed for conducting this review^3^.

### Literature search

Two study authors (GS and ST) performed the initial search of all the databases i.e., PubMed, Scopus, and Web of science. Databases were searched from their time of commencement until the last search conducted in March 2020. Following filters were applied “Human”, “English” and “randomized controlled trials”. The search strategy used for this review was based on Patient, Intervention, Control and Outcome (PICO) guideline. Following keywords and strategy were used:

*Patient* ureteric stone OR ureteric calculi OR ureterial stone OR ureteral calculi.

*Intervention* (ESWL) OR SWL) OR Shockwave lithotripsy) OR laparoscopic ureterolithotomy) OR laparoscopy ureterolithotomy) OR PNL) OR PCNL) OR percutaneous nephrolithotomy) OR ureteroscopy) OR ureterorenoscopy) OR URS.

*Outcome* initial and final stone free rate, duration of surgery, length of stay, complication rate.

Citation manager (EndNote) was used to populate the results obtained from the databases search, and the duplicates were removed. Bibliography of included articles and similar review articles were sought for additional articles. Search strategy used for PubMed has been provided in the supplementary file (S1).

### Study eligibility criteria

Following initial literature search, title and abstract screening were performed by the two review authors.

### Inclusion

1. A study should contain data for various surgical interventions on initial or final stone-free rate, need for auxiliary procedures, duration of surgery, length of hospital stay and complication rate for the management of large (> 1 cm) upper ureteric stones.
2. Only randomized control trials will be included in this review.

### Exclusion

1. Non-randomized studies
2. Case reports, editorials, letters and reviews.
3. Not containing data on initial or final stone-free rate, need for additional procedures, duration of surgery, length of hospital stay and complication rate for upper ureteric stone.
4. Studies comparing various surgical techniques with mean stone size less than 1 cm.
5. Studies in the pediatric age groups (age < 18 years) were excluded.

After title and abstract screening, studies satisfying inclusion criteria were included for full-text review. After a full-text review, studies were assessed for eligibility in the review. The study selection process was independently conducted by two authors, and the help of a third author was taken in case of discrepancy.

### Data extraction

Data from the included studies was extracted in a pre-determined format. Data for the following variables were obtained such as author name, year of publication, study type, country, type of surgical treatment, initial stone-free rate, final stone-free rate, need for auxiliary procedures, operative time, length of stay, age, sex, stone size, PNL sheath size, standard or mini PNL, flexible or semi-rigid URS and duration of follow up. Upon data extraction, matching was done for consistency in case of discrepancy rechecking of data was done.

### Outcome

In this study, the primary outcome studied was the final stone-free rate which was defined at variable time periods after surgery and using various modalities, including plain x-ray, ultrasound, or computed tomogram (CT). Similarly, initial stone-free rate may be defined at various intervals such as next day following surgery, 3 days after surgery or just prior to discharge. The efficacy of a surgical technique will be determined by the stone-free rates (initial and final). We also performed analysis for other secondary outcomes such as initial stone-free rate, duration of surgery, length of stay, and complications rates.

### Statistical analysis, risk of bias and quality of evidence

This NMA was performed using a ‘frequentist approach’ and was designed to compare various treatment groups for the primary and secondary outcomes. All the statistical analysis was performed using “Stata” (version 16; StataCorp, College Station, TX, USA)^4^ using “mvmeta” command^5^ and “network”^6^ and “network graph” packages^7^. Quality or certainty of the evidence was determined by using methodology as described by Salanti et al. ^8^ and Nikolakopoulou et al.^9^ using Confidence in Network Meta-analysis (CINeMA) web application for the primary outcome^10^ i.e., final stone-free rate. Further details are provided in the supplementary file section.

## Results

### Literature search and study characteristics

**Figure 1:**
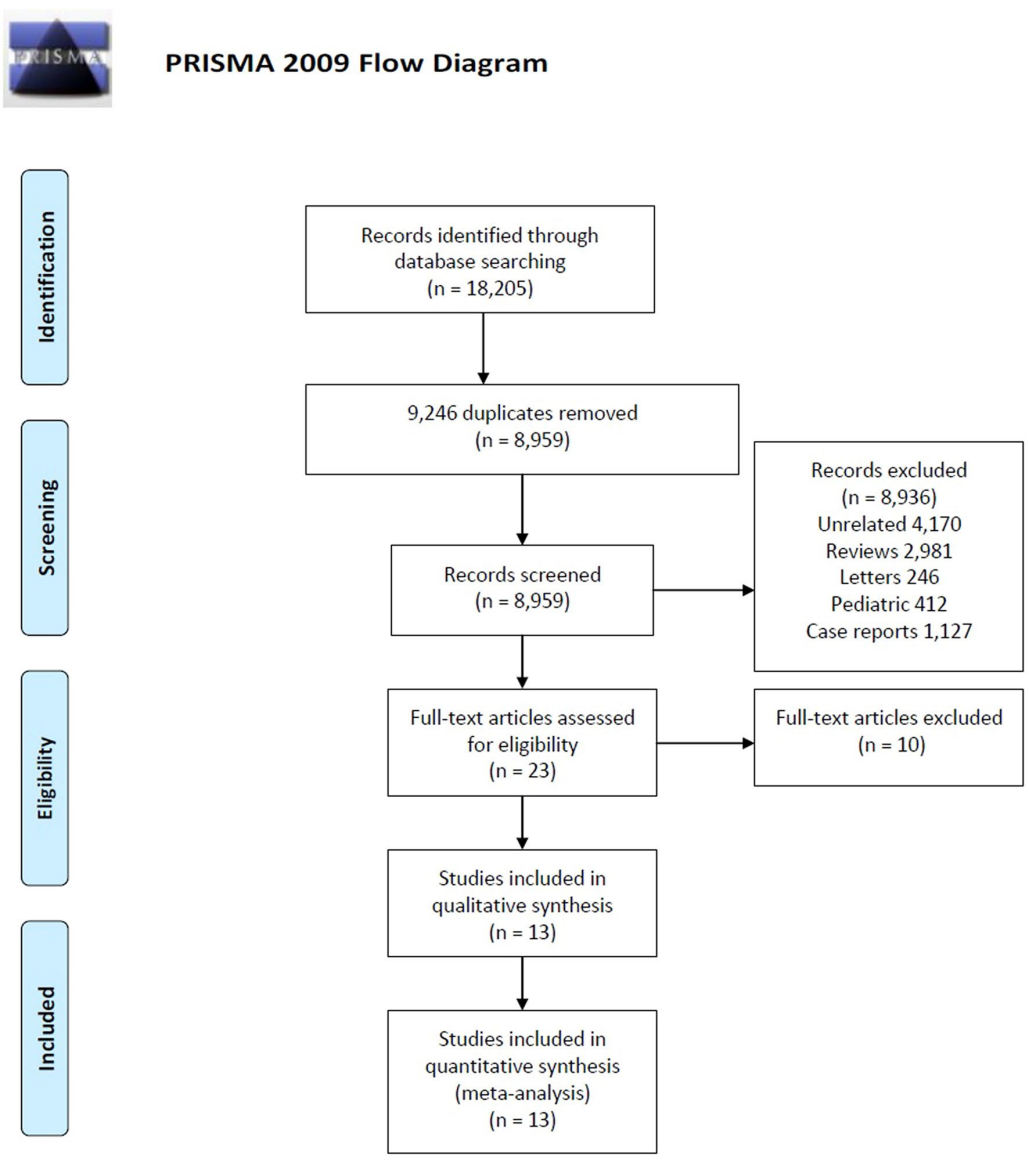
PRISMA flow-chart depicting search strategy used for conducting this study.

A literature search of electronic databases PubMed (4481), Scopus (10,538), Web of science (3186) returned 18,205 citations. Using the citation manager, 9246 duplicate references were removed. Another 8925 articles were excluded following initial title and abstract screening for not satisfying inclusion criteria (Fig. 1). Twenty-three articles underwent full text review, of which for final analysis, 13 articles were included. Remaining eight non-randomized studies and two studies with smaller stone size (< 1 cm) were excluded.

### Study characteristics

In this review, 13 RCTs with 1871 patients were included^11–22^. Four surgical treatments i.e., SWL, PNL, URS, and LUL, were compared for upper ureteric stones larger than 1 cm. Most of the studies included in this review were from Asia with 7 studies from China, 2 studies from India, 2 from Iran and one from Brazil and Turkey. Stone size cut-off was variable across different studies like 5 studies had stone size > 1.5 cm^12,17,19,22^, 3 studies had stone size > 1 cm^11,12,19^ and Kumar et al. had inclusion criteria of > 2 cm^22^ stone size. Semi-rigid ureteroscopy was the most commonly used technique for URS study except by Gu et al.^23^, who used both flexible and semi-rigid, and Ozturk et al.^17^, who used flexible URS. Laser lithotripsy was the most commonly used technique for URS lithotripsy except for Basiri et al.^18^, who used both pneumatic and laser, Lopes-Neto^11^ used pneumatic and Lee et al. used electrohydraulic and ultrasonic lithotripsy^13^. Among the techniques comparing PNL with other treatments, standard PNL^12,18^ was used in 2 studies, whereas mini-PNL in 4 studies. Among the techniques using LUL, retroperitoneal^19^ and transperitoneal^12,18,22^ approaches were used in 3 studies. Five studies were exclusively conducted on patients with impacted stones, whereas studies by Basiri et al.^18^ and Lopes-Neto et al.^11^ contained both impacted and non-impacted stones. The duration of follow-up was variable and has been shown in Table 1.

**Table 1:** Characteristics of the studies included in the overall analysis.

Author year country	Treatment group (n)	Age (years)	Sex (M/F)	Stone size (mm)	Type of ESWL device	Sheath size for PNL (French)	PNL	URS	Follow up (months)	Modality for follow up	Impaction	Modality for measuring stone size
Lopes-Neto 2012 Brazil	A-SWL(14) B-URS (16) C-LUL (15)	A-46 B-49.6 C-46	A-7/7 B-10/6 C-9/6	A-13.8 B-14.4 C-15.9	Dormeir compact delta S	–	Standard	Semirigid	2	X-ray KUB CT KUB	A-7.8% B-6.8% C-12.6%	NCCT KUB
Karami 2013 Iran	A-PNL (40) B-LUL (40)	A-39.4 B-35.2	A-28/12 B-24/16	A-14.2 B-13.5	NA	28–30	Standard	Rigid	6	USG KUB IVP	–	IVP
Lee 2006 China	A-SWL(22) B-URS (20)	A-54.2 ± 16.7 B-48.5 ± 13.3	A-19/3 B-16/4	A-17.9 ± 3.9 B-18.5 ± 2.9	Siemen AG Lithostar 2 Lithotripter	–	NA	NA	NA	X-ray KUB USG KUB IVP	–	IVP
Sun 2008 China	A-PNL(44) B-URS (47)	A-40.4 ± 8.4 B-39.6 ± 7.3	A-30/14 B-31/16	A-14.7 ± 2 B-14.6 ± 1.8	NA	14–16	Mini-PNL	Rigid	NA	X-ray KUB USG KUB	All	IVP
Wang 2017 China	A-URS (50) B-PNL(50) C-LUL(50)	A-42 ± 14 B-41 ± 15 C-44 ± 11	A-28/22 B-31/19 C-29/21	A-16.8 ± 2.1 B-19.3 ± 1.8 C-18.8 ± 1.4	NA	18	Mini-PNL	Rigid	12	X-ray KUB	All	Xray KUB
Gu 2012 China	A-PNL(30 B-URS (29)	A-42.5 ± 10.1 B-44.22 ± 13	A-1:0.81 B-1:0.64	A-17.3 B-16.2	NA	A-12–18 B-8.5/9.8	Mini-PNL	Semirigid	3	X-ray KUB USG KUB	All	Xray KUB/USG/NCCT
Yang 2012 China	A-PNL (91) B-URS (91)	A-45.2 ± 14.7 B-46.4 ± 15.1	A-53/38 B-54/37	A-17.9 B-15.8	NA	A-16 B-8/9.8	Mini-PNL	Rigid	12	X-ray KUB USG KUB	All	IVP
Ozturk 2013 Turkey	A-SWL (52) B- LUL (51) C- URS (48)	A-40.7 B-40.0 C-41.1	A-33/19 B-21/30 C-30/18	A-13.2 ± 2.04 B-13.3 ± 2.06 C-13.2 ± 2.01	Electrohydraulic extracorporeal Lithotripter (Multimed Classic)	–	–	Flexible	3	X-ray KUB USG KUB	–	–
Basiri 2008 Iran	A- URS (50) B- LUL (50) C- PNL (50)	A-39 ± 15 B-44 ± 13 C-48 ± 13	A-33/17 B-36/14 C-32/18	A-17.8 $\pm$ 2.4 A-22.4 $\pm$ 3.2 C-20.3 $\pm$ 3.3	–	–	Standard	Semirigid	3	X-ray KUB USG KUB	A-24 B-27 C-25	IVP
Fang 2012 China	A-URS (25) B—LUL(25)	A -36.9 ± 11.8 B-34.4 ± 9.8	A-15/10 B-14/11	A-15 ± 4 B-16 ± 3	–	–	–	Rigid		X-ray KUB	–	NCCT KUB
Shao 2015 China	A –URS (139) B-LUL (136)	A-41 B-40	A-90/49 B-92/44	A-13.6 $\pm$ 1.4 B-13.8 $\pm$ 1.9	–	–	–	Semirigid	A-20 B-20.5	NA	All	IVP/CT Urogram
Kumar 2015 India	A- LUL(50) B- URS (50)	A-36.7 ± 2.4 B-35.6 ± 2.1	A-24/26 B-26/24	A- 23 ± 2 B -22 ± 1	–	–	–	Semirigid	3	CT KUB	–	NCCT KUB
Kadyan 2016 India	A-URS (60) B-LUL (62)	A-44.3 $\pm 3.2$ B-42.1 $\pm 2.7$	A-38/22 B-37/25	A-16.8 $\pm$ 1.5 B-17.2 $\pm$ 1.9	NA	NA	NA	Semirigid	0.75	X-ray KUB USG KUB	–	–

PNL, Percutaneous Nephrolithotomy; URS, Uretero Renoscopic Lithotripsy; LUL, Laparoscopic Ureter lithotomy; SWL, Shock Wave Lithotripsy; KUB, Kidney Ureter Bladder; IVP, Intravenous Pyelography; CT, Computed Tomogram; USG, Ultra Sonography; NCCT, Non Contrast tomogram.

### Stone free rate

**Figure 2:**
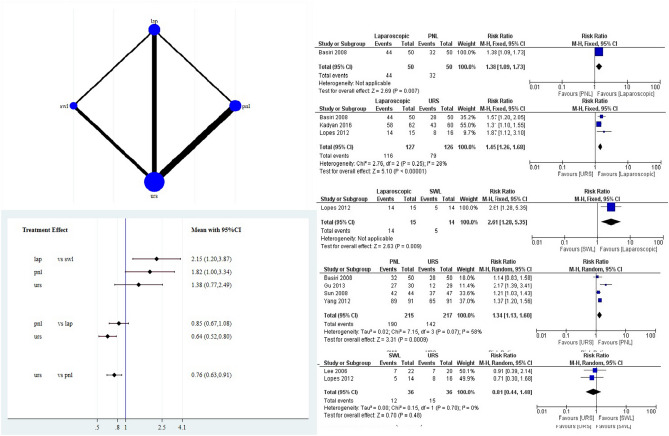
Depicts network map, interval plot and Forest plot for the initial stone free rates.

Data for the initial and final stone-free analysis was available from 7 and 12 studies, respectively. From the direct pair-wise comparison, LUL had a higher initial stone-free rate than PNL, URS, and SWL. Comparisons of LUL with PNL and LUL with SWL included data from a single study only. Compared to URS, PNL had a higher initial SFR. Lastly, URS and SWL had comparable initial SFR from direct pair-wise comparison. Pooled initial stone-free rates were 19.77%, 63.8%, 88.3%, and 91.3% for SWL, URS, PNL, and LUL, respectively. From network meta-analysis, for initial stone-free rate LUL and PNL were more effective than SWL. Also, PNL [risk ratio (RR) 0.76, 95% confidence interval (CI) (0.63, 0.91)] and LUL (RR 0.64, 95% CI (0.52, 0.80)) were better than URS. PNL and LUL were equally effective for initial SFR (RR 0.98, 95%CI (0.67, 1.08) (Fig. 2).

**Figure 3:**
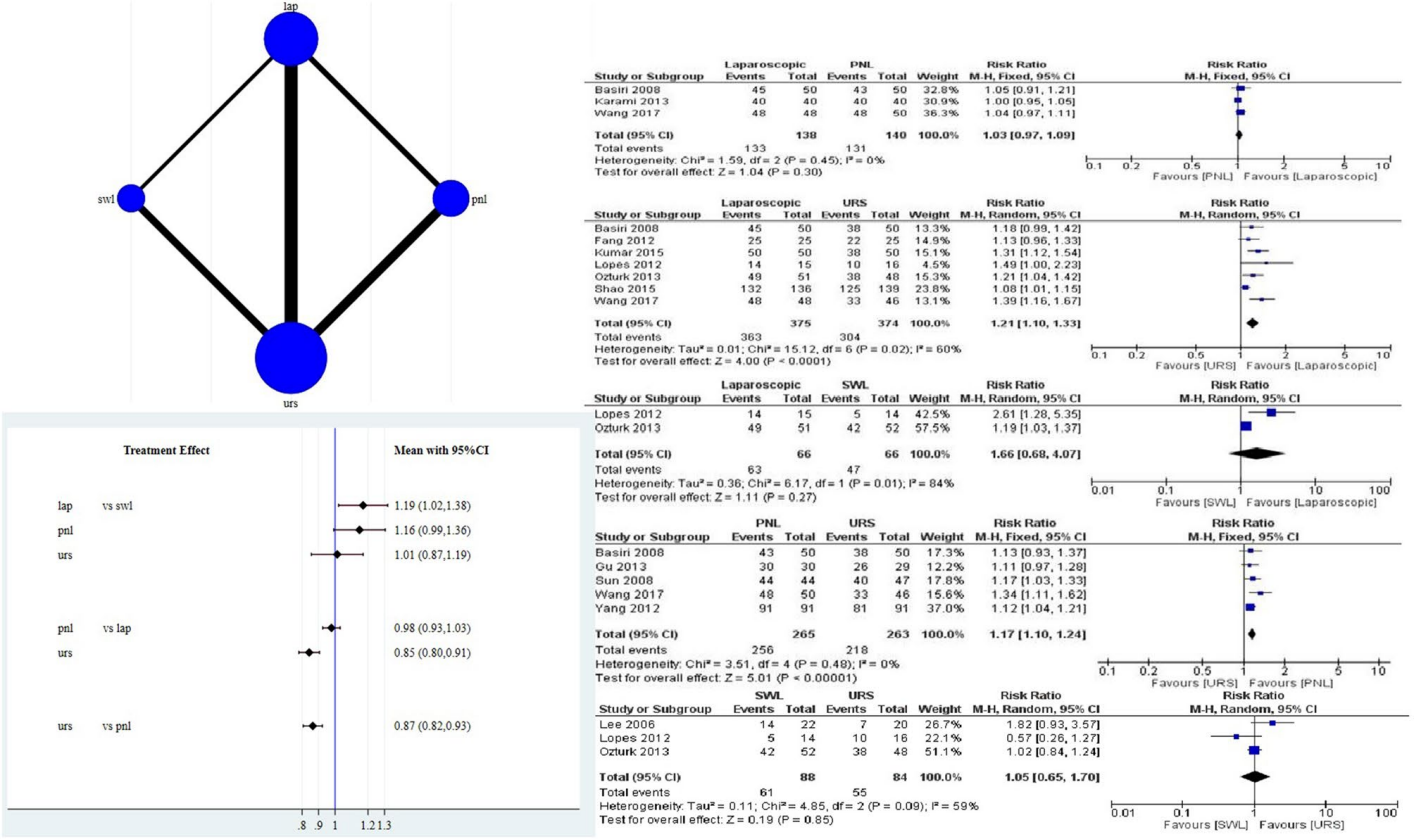
Depicts network map, interval plot and Forest plot for the final stone free rates.

For final stone-free rate analysis, direct comparison revealed PNL and LUL had similar final SFR (RR 1.03CI (0.97, 1.09) *p* = 0.30], whereas both PNL (RR 1.17 CI (1.10, 1.24) *p* =  < 0.00001], and LUL (RR 1.21 CI (1.10, 1.33) *p* = 0.0001] were significantly more effective than URS. However, the final stone-free rate for SWL and LUL ((RR 1.66 CI (0.68, 4.07) *p* = 0.27) were similar with pooled data from two studies only. SWL and URS also had similar final stone-free rate ((RR 1.05 CI (0.65, 1.70) *p* = 0.85). Pooled final stone-free rates were 72%, 81.5%, 96.7%, and 96.7% for SWL, URS, PNL, and LUL, respectively. From network meta-analysis, LUL (RR 1.21, 95%CI (1.07, 1.37)) had a higher final SFR than SWL. Whereas, PNL and SWL (RR 1.16, 95%CI (0.99, 1.336)) and; URS and SWL had similar final SFR (RR 1.04, 95%CI (0.92, 1.18)). URS had lower final SFR as compared to LUL (RR 0.85, 95%CI (0.80, 0.91)) and PNL (RR 0.87, 95%CI (0.82, 0.93)). LUL and PNL had similar final SFR (RR 0.98, 95%CI (0.93, 1.03)) (Fig. 3). For both initial and SFR, the surface under the cumulative ranking curve (SUCRA) values were highest for LUL, followed by PNL and URS (Fig. 3) (Table 2).

**Table 2:** Surface under cumulative ranking area (SUCRA) for all the treatment groups.

Treatment (SUCRA)	PNL (%)	LUL (%)	SWL (%)	URS (%)
Initial SFR	68.5	**97.1**	5.8	28.6
Final SFR	72.6	**92.7**	15.7	19
Auxiliary procedures	77.5	**88.3**	11.6	22.5
Duration of surgery	36.3	25.5	**84.7**	53.5
Length of stay	8.4	28.2	**85.8**	77.6
Complications	33.3	**73.2**	54.7	38.8

PNL, percutaneous nephrolithotomy; URS, ureterornoscopy; SEL, shockwave lithotripsy; SFR, stone free rate.

Bold signifies the highest SUCRA value in that category

### Auxiliary procedures

Data for auxiliary procedures were available from 9 studies. Direct pair-wise comparison revealed both PNL [RR 0.19 CI (0.05, 0.81) *p* < 0.02] and LUL [RR 0.20 CI (0.11, 0.37) *p* < 0.00001] needed a significantly lower number of auxiliary procedures compared to URS and the rest of the comparisons i.e., LUL with PNL, LUL with SWL and SWL with URS were not statistically significant. In network analysis, compared to SWL and URS, both PNL and LUL were associated with a significantly lower need for auxiliary procedures. Whereas, for LUL versus PNL (RR 1.29, 95%CI (0.36, 4.67)) and URS versus SWL ((RR 0.77, 95%CI (0.23, 2.59) there was no difference in the need for auxiliary procedures. Again for the need for auxiliary procedures, LUL was best followed by PNL and URS (Supplementary Figure 1 and Table 2).

### Duration of surgery and length of hospital stay

For the duration of surgery, data were available from 12 studies, standardized mean difference (SMD) for all the comparisons was not statistically significant, and a wide range of CI could be noted for both direct pairwise and network analysis except for one i.e., direct comparison of LUL vs. SWL. Data for comparison of SWL with LUL was available from a single study and favored SWL. According to SUCRA values, SWL (84.7) was the best treatment, followed by URS (53.5) (Supplementary Figure 2 and Table 2).

Length of hospital stay was significantly shorter with URS as compared to LUL [SMD 1.84 CI (0.27, 3.42) *p* = 0.02] and PNL [SMD 4.1 CI (2.0, 6.1) *p* = 0.0001] on direct comparison. Furthermore, SWL had a shorter length of stay than LUL and URS based on data from a single study. Length of stay was similar between PNL and LUL [SMD 0.03 CI (− 1.04, 1.1) *p* = 0.96] from direct comparison. From network analysis, there was no difference noted for the following comparisons LUL versus SWL, LUL versus PNL, PNL versus SWL, and URS versus SWL. However, compared to PNL (SMD − 3.36, 95%CI (− 5.5, − 1.2)) and LUL (SMD − 2.4, 95%CI (− 4.2, − 0.7)), URS was associated with a shorter length of hospital stay. According to SUCRA values, treatment groups were ranked as SWL > URS > LUL > PNL (Supplementary Fig. 3 and Table 2).

### Complications rate

Complication rates were not explicitly reported in all the studies. Some studies reported in standard Clavien-Dindo format while others did not. Initially, we planned to provide subgroup analysis according to bleeding complications or major and minor complications, but the same was not possible due to lack of data consistency across the studies. Overall complication rates were not significantly different across various comparisons for both direct pairwise and network analysis (Supplementary Fig. 4) and, according to SUCRA, they were ranked as LUL > SWL > URS > PNL (Supplementary Fig. 4 and Table 2).

### Inconsistency

Inconsistency, as evaluated using ‘global’ and ‘node-splitting’ approach did not reveal any evidence of inconsistency for all the comparisons for all the outcomes. Heterogeneity was also estimated using loop approach (Supplementary Fig. 5) and revealed no inconsistency.

### Risk of bias and certainty of evidence

Certainty of evidence was assessed using the CINeMA approach for all the comparisons for the primary outcome i.e., the final stone-free rate. The confidence rating was between ‘very low’ to ‘moderate’ for the comparisons (Supplementary Table 1). Visual assessment of the Funnel plot revealed symmetry, thus indicating a lack of publication bias (Supplementary Fig. 6). Details are provided in the supplementary file.

## Discussion

For the management of upper ureteric stones, SWL, URS, PNL, and LUL are the available options, each associated with its own success rates and morbidities. For upper ureteric stones of > 1 cm size, URS (retrograde or antegrade) and SWL have been suggested as the recommended treatment modalities for treating large upper ureteric calculi in various guidelines^24,25^. The same can be attributed to acceptable stone clearance rates with minimal morbidity. PNL is a relatively more invasive procedure with the inherent risk of bleeding, is not considered the first line approach for treating upper ureteric stones. However, PNL can be preferred in certain situations like large impacted ureteric stone, associated renal stones, situations where retrograde ureteroscopic access is difficult, like stones in transplanted kidneys and patients on urinary diversion. Among all the surgical modalities, LUL has the best stone clearance rates after a single procedure^26^ for the treatment of the large upper ureteric calculi. Its perceived invasive nature could explain apprehension regarding its use for upper ureteric stones. Even in the guidelines, LUL is still considered an optional treatment modality or in cases where other treatment modalities have failed or unlikely to succeed. With this study, we aimed to generate data from network analysis to determine the most efficacious and least morbid procedure for treating large upper ureteric stones.

Complete stone clearance is the definitive goal of all the treatment modalities, which is dependent upon multiple factors. To begin with, stone size is an essential factor that can limit the efficacy of SWL, URS to a certain extent but not for LUL and PNL(antegrade). This could explain decreased efficacy (low initial and final SFRs) of SWL and URS compared to LUL and PNL, as noted in our study. Stone impaction is another factor limiting the efficacy of SWL and URS but not PNL and LUL. Some studies in this meta-analysis had specifically included this group of patients, but their overall number was small; hence subgroup analysis was omitted. Other stone-related factors affecting clearance rates are stone composition and density which were not adequately stratified across the studies. In the present study, we noted LUL to be the most efficacious treatment modality for large upper ureteric stones compared to others. Enbloc stone removal irrespective of stone size, composition, and impaction could be the reason for this higher efficacy.

Need auxiliary treatment for residual stones is another important parameter that has significant financial and psychological implications. Our study revealed that LUL and PNL needed a lower number of auxiliary treatments than URS and SWL. According to the SUCRA values, LUL was the best treatment, followed by PNL for the need of auxiliary treatment. This could be attributed to higher initial stone-free rates for LUL, thus requiring fewer adjunctive treatments. In a previous meta-analysis by Kallidonis et al.^27^, comparing LUL with URS for authors noted higher stone free rates (initial and final) and lower need for secondary treatments for LUL group for upper ureteric stones. Similar results were also noted by Toricelli et al.^28^ in their meta-analysis comparing LUL with URS for upper ureter stones. In a meta-analysis by Wang et al.^29^ comparing URS with PNL for upper ureteric stone, higher stone-free rates (initial and final) and lower need for auxiliary treatments were noted for the PNL group. However, both of the above-mentioned meta-analyses were not specifically done for large upper ureteric stones.

For the duration of surgery, previous studies by Kallidonis^27^ and Torricelli et al.^28^ have reported shorter operative time with URS compared to LUL for upper ureteric stones. Wang et al.^29^ also reported shorter operative time for URS compared to PNL. On the contrary, we did not find any significant difference between any of the treatment modalities for lafre upper ureteric stones in the present study. Hence, from our results, we can state that duration of surgery should not be a deciding factor while considering any treatment modality.

Our study’s, length of stay was shorter for URS than both LUL and PNL from both direct and network analyses. It is to be noted that length of stay depends on multiple factors such as intra-operative or post-operative complications, institutional policy, surgeon preferences ,and insurance driven. Moreover, most of the centers around the world perform SWL and URS as daycare procedures unless the clinical situation demands (severe renal colic following SWL, ureter injury during URS or bleeding following any procedure). Thus, the relevance of this component seems to be very little from this meta-analysis. However, in a given situation, a patient may prefer a daycare procedure over a lengthy hospital stay. However, this should never be the main force driving clinical decision-making. Like our study, URS has been favorable compared to LUL and PNL for the length of stay in the previous studies^33^.

In the present study, overall complication rates were similar across all the comparisons for both direct as well network meta-analyses. However, according to the SUCRA values obtained, LUL was the best treatment with the least complications, followed by SWL. The better safety profile of LUL irrespective of the stone size compared to other treatment modalities like URS, SWL and PNL could be the reason for this observation. Hence, despite being perceived as a more invasive treatment option, LUL has a better morbidity profile than other modalities for large upper ureteric stones. Each of these surgical techniques involves a different surgical approach and thus unique complications. The laparoscopic procedure can lead to a higher need for analgesia and postoperative ileus, more so for the transperitoneal approach. PNL entails the penetration of renal parenchyma; thus bleeding remains the most worrisome complication associated with the procedure. Few complications specific to SWL include perirenal hematoma, contusions, and obstruction due to stone fragments. We also acknowledge that we did not perform subgroup analysis according to major and minor complications as the data for same was available from few studies only. Study authors have encountered similar problems in previous meta-analyses such as by Kallidonis et al.^27^ who acknowledged about the lack of data in uniform format that precluded formal quantitative analyses thus providing only qualitative data. Similarly, Wang et al.^29^ could provide quantitative data for individual complications such as fever, ureteral perforation, and blood transfusion.

Most of the studies in this review have been plagued by allocation, detection and performance biases. Confidence for each comparison was assessed using the CINeMA approach and three comparisons were rated “very low”, one as “low” and two as “moderate”. Hence, further high-quality prospective RCTs are needed comparing different treatment modalities for the management of large upper ureteric stones.

## Limitations

There are certain limitations of this meta-analysis that should be acknowledged. Firstly, the quality of studies included in this review is of concern which has been highlighted previously. Secondly, there was heterogeneity in the studies for some important parameters such as stone size, duration of follow up and modality used to determine the stone-free status. Thirdly, there was heterogeneity among individual surgical techniques such as type of device used for SWL, energy modality used for URS (laser or pneumatic or electrohydraulic), flexible or rigid URS, standard or mini-PNL, and retroperitoneal or laparoscopic approach used for LUL. Heterogeneity in initial modality (such as CT, USG and IVP) used for measuring the stone size and similar heterogeneity in the follow-up modalities makes the interpretation of results difficult. Fourthly, we initially intended to perform sensitivity analysis according to stone size, type of energy device used for URS, type of approach used for LUL and PNL. However, the same could not be possible as very small number of studies were left for analyses. Lastly, from the methodology point of view, we acknowledge the exclusion of conference abstracts and limiting the literature search to English. However, our literature search was extensive as well as thorough. We also note that the ‘frequentist’ approach used for conducting this NMA has its own limitationsy^2^.

## Conclusion

Laparoscopic ureterolithotomy followed by antegrade PNL is the most efficacious modality for the management of large upper ureteric stones with superior stone-free rates and lesser need for auxiliary treatments as compared to SWL and URS. However, the results of this study have been plagued by the poor quality of included studies. Thus the choice of a treatment modality in a given situation will continue to depend upon patient and surgeon-driven factors until a good-quality randomized controlled trial comparing all the four modalities for large upper ureteric stones comes up.

## Supplementary Information

Supplementary Information.

## References

[1] Türk, C., Neisius, A., Petřík, A., Seitz, C., Thomas, K. & Skolarikos, A. EAU Guidelines on Urolithiasis 2020. European Association of Urology Guidelines. 2020 Edition. presented at the EAU Annual Congress Amsterdam 2020. The European Association of Urology Guidelines Office; 2020.

[2] Shim S, Yoon BH, Shin IS, Bae JM. Network meta-analysis: application and practice using Stata. Epidemiol Health. 2017;39:e2017047. 10.4178/epih.e2017047 PMC5733388 29092392

[3] Hutton B, Salanti G, Caldwell DM, Chaimani A, Schmid CH, Cameron C (2015). The PRISMA extension statement for reporting of systematic reviews incorporating network meta-analyses of health care interventions: checklist and explanations. Ann. Intern. Med..

[4] StataCorp. Stata statistical software: release 16. StataCorp LLC (2019).

[5] White IR, Barrett JK, Jackson D, Higgins JP (2012). Consistency and inconsistency in network meta-analysis: model estimation using multivariate meta-regression. Res. Synth. Methods.

[6] White I (2015). Network meta-analysis. Stata J..

[7] Chaimani A, Higgins JP, Mavridis D, Spyridonos P, Salanti G (2013). Graphical tools for network meta-analysis in STATA. PLoS ONE.

[8] Salanti G, Del Giovane C, Chaimani A, Caldwell DM, Higgins JP (2014). Evaluating the quality of evidence from a network meta-analysis. PLoS ONE.

[9] Nikolakopoulou A, Higgins JPT, Papakonstantinou T, Chaimani A, Del Giovane C, Egger M (2020). CINeMA: an approach for assessing confidence in the results of a network meta-analysis. PLoS Med..

[10] Tiloklurs C, Taweemonkongsap T, Amornvesukit T, Phinthusophon K, Nualyong C, Chotikawanich E (2017). Comparison of successful treatment between ureteroscopic lithotripsy and extracorporeal shock wave lithotripsy for proximal ureteric calculi. J. Med. Assoc. Thailand.

[11] Lopes Neto AC, Korkes F, Silva Ii JL, Amarante RDM, Mattos MHE, Tobias-Machado M (2012). Prospective randomized study of treatment of large proximal ureteral stones: extracorporeal shock wave lithotripsy versus ureterolithotripsy versus laparoscopy. J. Urol..

[12] Karami H, Mazloomfard MM, Lotfi B, Alizadeh A, Javanmard B (2013). Ultrasonography-guided PNL in comparison with laparoscopic ureterolithotomy in the management of large proximal ureteral stone. Int. Braz. J..

[13] Lee YH, Tsai JY, Jiaan BP, Wu T, Yu CC (2006). Prospective randomized trial comparing shock wave lithotripsy and ureteroscopic lithotripsy for management of large upper third ureteral stones. Urology.

[14] Sun X, Xia S, Lu J, Liu H, Han B, Li W (2008). Treatment of large impacted proximal ureteral stones: randomized comparison of percutaneous antegrade ureterolithotripsy versus retrograde ureterolithotripsy. J. Endourol. Endourol. Soc.y..

[15] Wang Y, Zhong B, Yang X, Wang G, Hou P, Meng J (2017). Comparison of the efficacy and safety of URSL, RPLU, and MPCNL for treatment of large upper impacted ureteral stones: a randomized controlled trial. BMC Urol..

[16] Yang Z, Song L, Xie D, Hu M, Peng Z, Liu T (2012). Comparative study of outcome in treating upper ureteral impacted stones using minimally invasive percutaneous nephrolithotomy with aid of patented system or transurethral ureteroscopy. Urology.

[17] Ozturk U, Can Şener N, Goksel Goktug HN, Gucuk A, Nalbant I, Abdurrahim IM (2013). The comparison of laparoscopy, shock wave lithotripsy and retrograde intrarenal surgery for large proximal ureteral stones. J. Can. Urol. Assoc..

[18] Basiri A, Simforoosh N, Ziaee A, Shayaninasab H, Moghaddam S, Zare S (2008). Retrograde, antegrade, and laparoscopic approaches for the management of large, proximal ureteral stones: a randomized clinical trial. J. Endourol..

[19] Fang Y, Qiu J, Wang D, Zhan H, Situ J (2012). Comparative study on ureteroscopic lithotripsy and laparoscopic ureterolithotomy for treatment of unilateral upper ureteral stones. Acta Cirurgica Bras..

[20] Shao Y, Wang DW, Lu GL, Shen ZJ (2015). Retroperitoneal laparoscopic ureterolithotomy in comparison with ureteroscopic lithotripsy in the management of impacted upper ureteral stones larger than 12 mm. World J. Urol..

[21] Kadyan B, Sabale VP, Kankalia SP, Satav V, Jain DK, Mulay A (2015). A Prospective randomized study of large proximal ureteral stones: ureterolithotripsy versus laparoscopy. Indian J. Urol..

[22] Kumar A, Vasudeva P, Nanda B, Kumar N, Jha SK, Singh H (2015). A prospective randomized comparison between laparoscopic ureterolithotomy and semirigid ureteroscopy for upper ureteral stones >2cm: a single-center experience. J. Endourol. Endourol. Soc..

[23] Xiao-jian G, Jian Lin L, Yan X (2013). Treatment of large impacted proximal ureteral stones: randomized comparison of minimally invasive percutaneous antegrade ureterolithotripsy versus retrograde ureterolithotripsy. World J. Urol..

[24] Türk C, Petřík A, Sarica K, Seitz C, Skolarikos A, Straub M (2016). EAU guidelines on interventional treatment for urolithiasis. Eur. Urol..

[25] Preminger GM, Tiselius HG, Assimos DG, Alken P, Buck C, Gallucci M (2007). 2007 Guideline for the management of ureteral calculi. J. Urol..

[26] Keeley FX, Gialas I, Pillai M, Chrisofos M, Tolley DA (1999). Laparoscopic ureterolithotomy: the Edinburgh experience. Bju Int..

[27] Kallidonis P, Ntasiotis P, Knoll T, Sarica K, Papatsoris A, Somani BK (2017). Minimally invasive surgical ureterolithotomy versus ureteroscopic lithotripsy for large ureteric stones: a systematic review and meta-analysis of the literature. Eur. Urol. Focus.

[28] Torricelli FC, Monga M, Marchini GS, Srougi M, Nahas WC, Mazzucchi E (2016). Semi-rigid ureteroscopic lithotripsy versus laparoscopic ureterolithotomy for large upper ureteral stones: a meta-analysis of randomized controlled trials. Int. Braz. J. Urol..

[29] Wang Q, Guo J, Hu H, Lu Y, Zhang J, Qin B (2017). Rigid ureteroscopic lithotripsy versus percutaneous nephrolithotomy for large proximal ureteral stones: a meta-analysis. PLoS ONE.

