# Supplementary material for: Comparison of efficacy and safety of various management options for large upper ureteric stones a systematic review and network meta-analysis
Source: Sci Rep. 2021 Jun 3;11:11811. doi: 10.1038/s41598-021-91364-3 (PMC8175352; doi:10.1038/s41598-021-91364-3)
Supplement: Supplementary file 1 — Supplementary Information. [file 41598_2021_91364_MOESM1_ESM.docx]

**Title:** Comparison of efficacy and safety of various management options for large upper ureteric stones a systematic review and network meta-analysis.

**Running Title:** Large upper ureter stone: various surgical options

**AUTHORS**

1. Dr. Gopal Sharma MS

Senior Resident, Dept. of Urology

PGIMER, Chandigarh

Email: [gopal.26669192@gmail.com](mailto:gopal.26669192@gmail.com)

1. Dr. Tarun Pareek MS

Senior Resident, Dept. of Urology

PGIMER, Chandigarh

Email: [maverickpareek@gmail.com](mailto:maverickpareek@gmail.com)

1. Dr. Shantanu Tyagi MS

Senior Resident, Dept. of Urology

PGIMER, Chandigarh

Email: [metyagishantanu@gmail.com](mailto:metyagishantanu@gmail.com)

1. Dr. Pawan Kaundal MS

Senior Resident, Dept. of Urology

PGIMER, Chandigarh

Email: [drpawan19@gmail.com](mailto:drpawan19@gmail.com)

1. Dr. Anuj Kumar Yadav MS

Senior Resident, Dept. of Urology

PGIMER, Chandigarh

Email: [asisotia@gmail.com](mailto:asisotia@gmail.com)

1. Dr. Yashasvi Thummala MS

Senior Resident, Dept. of Urology

PGIMER, Chandigarh

Email: [yashaswireddy1@gmail.com](mailto:yashaswireddy1@gmail.com)

1. Dr. Sudheer Kumar Devana MCh

Associate Professor, Dept. of Urology

PGIMER, Chandigarh

Email: [drsudheer1983@gmail.com](mailto:drsudheer1983@gmail.com)

**Institutional affiliation**

1. Dept. of Urology, Postgraduate Institute of Medical Education and Research, Chandigarh, India

**Previous presentation in conferences**: None

**Conflict of interest:** None to declare

**Funding:** None to declare

**Acknowledgments:** None

***Corresponding Author**

Dr. Sudheer Kumar Devana MCh

Associate Professor, Dept. of Urology

PGIMER, Chandigarh

Email: [drsudheer1983@gmail.com](mailto:drsudheer1983@gmail.com)

ADDRESS: LEVEL II, B BLOCK, ADVANCED UROLOGY CENTER, PGIMER, SECTOR 12, CHANDIGARH 160012.

CONTACT NO: (+91) 9855509868

Word count: 3786

Table count: 2

Figure Count: 4

**Supplementary Table 1:** Search strategy used for PubMed

| \| Query \| Search Details \| Results \| \| --- \| --- \| --- \| \| ((((ureteral stone) OR (ureteral calculi)) OR (ureteric calculi)) OR (ureteric stone)) AND (((((((((((URS) OR (ureterorenoscopy)) OR (ureteroscopy)) OR (percutaneous nephrolithotomy)) OR (PCNL)) OR (PNL)) OR (laparoscopy ureterolithotomy)) OR (laparoscopic ureterolithotomy)) OR (Shockwave lithotripsy)) OR (SWL)) OR (ESWL)) \| ("ureterolithiasis"[MeSH Terms] OR "ureterolithiasis"[All Fields] OR ("ureteral"[All Fields] AND "stone"[All Fields]) OR "ureteral stone"[All Fields] OR "ureteral calculi"[MeSH Terms] OR ("ureteral"[All Fields] AND "calculi"[All Fields]) OR "ureteral calculi"[All Fields] OR ("ureteral"[All Fields] AND "stone"[All Fields]) OR ("ureteral calculi"[MeSH Terms] OR ("ureteral"[All Fields] AND "calculi"[All Fields]) OR "ureteral calculi"[All Fields]) OR (("ureter"[MeSH Terms] OR "ureter"[All Fields] OR "ureteral"[All Fields] OR "ureteric"[All Fields] OR "ureteritis"[All Fields]) AND ("calculi"[MeSH Terms] OR "calculi"[All Fields] OR "calculis"[All Fields])) OR ("ureterolithiasis"[MeSH Terms] OR "ureterolithiasis"[All Fields] OR ("ureteric"[All Fields] AND "stone"[All Fields]) OR "ureteric stone"[All Fields] OR "ureteral calculi"[MeSH Terms] OR ("ureteral"[All Fields] AND "calculi"[All Fields]) OR "ureteral calculi"[All Fields] OR ("ureteric"[All Fields] AND "stone"[All Fields]))) AND ("URS"[All Fields] OR ("ureterorenoscopies"[All Fields] OR "ureterorenoscopy"[All Fields]) OR ("ureteroscopy"[MeSH Terms] OR "ureteroscopy"[All Fields] OR "ureteroscopies"[All Fields]) OR ("nephrolithotomy, percutaneous"[MeSH Terms] OR ("nephrolithotomy"[All Fields] AND "percutaneous"[All Fields]) OR "percutaneous nephrolithotomy"[All Fields] OR ("percutaneous"[All Fields] AND "nephrolithotomy"[All Fields])) OR "PCNL"[All Fields] OR "PNL"[All Fields] OR (("laparoscopie"[All Fields] OR "laparoscopy"[MeSH Terms] OR "laparoscopy"[All Fields] OR "laparoscopies"[All Fields]) AND ("ureterolithotomies"[All Fields] OR "ureterolithotomy"[All Fields])) OR (("laparoscopes"[MeSH Terms] OR "laparoscopes"[All Fields] OR "laparoscope"[All Fields] OR "laparoscopical"[All Fields] OR "laparoscopically"[All Fields] OR "laparoscopics"[All Fields] OR "laparoscopy"[MeSH Terms] OR "laparoscopy"[All Fields] OR "laparoscopic"[All Fields]) AND ("ureterolithotomies"[All Fields] OR "ureterolithotomy"[All Fields])) OR (("shockwave"[All Fields] OR "shockwaves"[All Fields]) AND ("lithotripsy"[MeSH Terms] OR "lithotripsy"[All Fields] OR "lithotripsies"[All Fields])) OR "SWL"[All Fields] OR "ESWL"[All Fields]) \| 4,481 \| \| (((ureteral stone) OR (ureteral calculi)) OR (ureteric calculi)) OR (ureteric stone) \| "ureterolithiasis"[MeSH Terms] OR "ureterolithiasis"[All Fields] OR ("ureteral"[All Fields] AND "stone"[All Fields]) OR "ureteral stone"[All Fields] OR "ureteral calculi"[MeSH Terms] OR ("ureteral"[All Fields] AND "calculi"[All Fields]) OR "ureteral calculi"[All Fields] OR ("ureteral"[All Fields] AND "stone"[All Fields]) OR ("ureteral calculi"[MeSH Terms] OR ("ureteral"[All Fields] AND "calculi"[All Fields]) OR "ureteral calculi"[All Fields]) OR (("ureter"[MeSH Terms] OR "ureter"[All Fields] OR "ureteral"[All Fields] OR "ureteric"[All Fields] OR "ureteritis"[All Fields]) AND ("calculi"[MeSH Terms] OR "calculi"[All Fields] OR "calculis"[All Fields])) OR ("ureterolithiasis"[MeSH Terms] OR "ureterolithiasis"[All Fields] OR ("ureteric"[All Fields] AND "stone"[All Fields]) OR "ureteric stone"[All Fields] OR "ureteral calculi"[MeSH Terms] OR ("ureteral"[All Fields] AND "calculi"[All Fields]) OR "ureteral calculi"[All Fields] OR ("ureteric"[All Fields] AND "stone"[All Fields])) \| 11,977 \| \| ((((((((((URS) OR (ureterorenoscopy)) OR (ureteroscopy)) OR (percutaneous nephrolithotomy)) OR (PCNL)) OR (PNL)) OR (laparoscopy ureterolithotomy)) OR (laparoscopic ureterolithotomy)) OR (Shockwave lithotripsy)) OR (SWL)) OR (ESWL) \| "URS"[All Fields] OR ("ureterorenoscopies"[All Fields] OR "ureterorenoscopy"[All Fields]) OR ("ureteroscopy"[MeSH Terms] OR "ureteroscopy"[All Fields] OR "ureteroscopies"[All Fields]) OR ("nephrolithotomy, percutaneous"[MeSH Terms] OR ("nephrolithotomy"[All Fields] AND "percutaneous"[All Fields]) OR "percutaneous nephrolithotomy"[All Fields] OR ("percutaneous"[All Fields] AND "nephrolithotomy"[All Fields])) OR "PCNL"[All Fields] OR "PNL"[All Fields] OR (("laparoscopie"[All Fields] OR "laparoscopy"[MeSH Terms] OR "laparoscopy"[All Fields] OR "laparoscopies"[All Fields]) AND ("ureterolithotomies"[All Fields] OR "ureterolithotomy"[All Fields])) OR (("laparoscopes"[MeSH Terms] OR "laparoscopes"[All Fields] OR "laparoscope"[All Fields] OR "laparoscopical"[All Fields] OR "laparoscopically"[All Fields] OR "laparoscopics"[All Fields] OR "laparoscopy"[MeSH Terms] OR "laparoscopy"[All Fields] OR "laparoscopic"[All Fields]) AND ("ureterolithotomies"[All Fields] OR "ureterolithotomy"[All Fields])) OR (("shockwave"[All Fields] OR "shockwaves"[All Fields]) AND ("lithotripsy"[MeSH Terms] OR "lithotripsy"[All Fields] OR "lithotripsies"[All Fields])) OR "SWL"[All Fields] OR "ESWL"[All Fields] \| 17,831 \| \| URS \| "URS"[All Fields] \| 2,828 \| \| ureterorenoscopy \| "ureterorenoscopies"[All Fields] OR "ureterorenoscopy"[All Fields] \| 807 \| \| ureteroscopy \| "ureteroscopy"[MeSH Terms] OR "ureteroscopy"[All Fields] OR "ureteroscopies"[All Fields] \| 5,668 \| \| percutaneous nephrolithotomy \| "nephrolithotomy, percutaneous"[MeSH Terms] OR ("nephrolithotomy"[All Fields] AND "percutaneous"[All Fields]) OR "percutaneous nephrolithotomy"[All Fields] OR ("percutaneous"[All Fields] AND "nephrolithotomy"[All Fields]) \| 4,158 \| \| PCNL \| "PCNL"[All Fields] \| 2,161 \| \| PNL \| "PNL"[All Fields] \| 1,931 \| \| laparoscopy ureterolithotomy \| ("laparoscopie"[All Fields] OR "laparoscopy"[MeSH Terms] OR "laparoscopy"[All Fields] OR "laparoscopies"[All Fields]) AND ("ureterolithotomies"[All Fields] OR "ureterolithotomy"[All Fields]) \| 195 \| \| laparoscopic ureterolithotomy \| ("laparoscopes"[MeSH Terms] OR "laparoscopes"[All Fields] OR "laparoscope"[All Fields] OR "laparoscopical"[All Fields] OR "laparoscopically"[All Fields] OR "laparoscopics"[All Fields] OR "laparoscopy"[MeSH Terms] OR "laparoscopy"[All Fields] OR "laparoscopic"[All Fields]) AND ("ureterolithotomies"[All Fields] OR "ureterolithotomy"[All Fields]) \| 241 \| \| Shockwave lithotripsy \| ("shockwave"[All Fields] OR "shockwaves"[All Fields]) AND ("lithotripsy"[MeSH Terms] OR "lithotripsy"[All Fields] OR "lithotripsies"[All Fields]) \| 2,885 \| \| SWL \| "SWL"[All Fields] \| 1,562 \| \| ESWL \| "ESWL"[All Fields] \| 3,055 \| \| ureteral stone \| "ureterolithiasis"[MeSH Terms] OR "ureterolithiasis"[All Fields] OR ("ureteral"[All Fields] AND "stone"[All Fields]) OR "ureteral stone"[All Fields] OR "ureteral calculi"[MeSH Terms] OR ("ureteral"[All Fields] AND "calculi"[All Fields]) OR "ureteral calculi"[All Fields] OR ("ureteral"[All Fields] AND "stone"[All Fields]) \| 10,601 \| \| ureteral calculi \| "ureteral calculi"[MeSH Terms] OR ("ureteral"[All Fields] AND "calculi"[All Fields]) OR "ureteral calculi"[All Fields] \| 9,178 \| \| ureteric calculi \| ("ureter"[MeSH Terms] OR "ureter"[All Fields] OR "ureteral"[All Fields] OR "ureteric"[All Fields] OR "ureteritis"[All Fields]) AND ("calculi"[MeSH Terms] OR "calculi"[All Fields] OR "calculis"[All Fields]) \| 10,508 \| \| ureteric stone \| "ureterolithiasis"[MeSH Terms] OR "ureterolithiasis"[All Fields] OR ("ureteric"[All Fields] AND "stone"[All Fields]) OR "ureteric stone"[All Fields] OR "ureteral calculi"[MeSH Terms] OR ("ureteral"[All Fields] AND "calculi"[All Fields]) OR "ureteral calculi"[All Fields] OR ("ureteric"[All Fields] AND "stone"[All Fields]) \| 10,002 \| |
| --- | --- | --- | --- | --- | --- | --- | --- | --- | --- | --- | --- | --- | --- | --- | --- | --- | --- | --- | --- | --- | --- | --- | --- | --- | --- | --- | --- | --- | --- | --- | --- | --- | --- | --- | --- | --- | --- | --- | --- | --- | --- | --- | --- | --- | --- | --- | --- | --- | --- | --- | --- | --- | --- | --- | --- | --- | --- |

**Statistical analysis**

For direct pair wise comparison of continuous outcomes, data was calculated as mean difference (MD) together with their 95% confidence interval (95% CI). Mean and standard deviation was estimated from the median and range using the formula reported by Hozo et al[^1^](#_ENREF_1) wherever they were missing. For dichotomous variables data was assessed using risk ratios. Statistical heterogeneity was tested using chi^2^ and I^2^ tests. A p value < 0.10 was used to indicate heterogeneity and in the absence of statistical heterogeneity the fixed-effects model (Mantel-Haenszel method) was used. In the presence of statistically significant heterogeneity random effects model was used. A p-value of <0.05 indicates statistical significance. For direct pair wise comparison, statistical analysis was performed using the Cochrane collaboration review manager software RevMan 5.2^TM^ (the Cochrane collaboration, Copenhagen, Denmark). Network meta-analysis was aimed to combine both direct and indirect evidence into a single effect size for all the comparisons i.e. surgical modalities for all the outcomes (initial and final stone free rates, need for auxiliary procedures, complication rates, duration of hospital stay and duration of surgery). We presented estimated mean differences in with their 95% confidence interval (CI) in interval plots and league tables for all the comparisons. Relative rankings of various surgical treatments were estimated for all the outcomes using the distribution of ranking probabilities and surface under the cumulative ranking curves (SUCRA). For publication bias visual interpretation of comparison adjusted forest plots was done. All the statistical analysis was performed using Stata (version 16; StataCorp, College Station, TX, USA)[^2^](#_ENREF_2) using “network[^3^](#_ENREF_3)” and “network graph” packages[^4^](#_ENREF_4).

**Inconsistency**

Inconsistency refers to existence of difference between direct and indirect estimates for a given comparison and presence of inconsistency can jeopardizes the validity of the results. Inconsistency evaluation was done using both global and local approaches. In global approach for inconsistency, first level of inconsistency is computed according to between-treatment comparison for all cases followed by test for global linearity using Wald test. In local approaches using node splitting each treatment is individually examined and outcomes of direct and indirect comparisons are tested. Loops specific approach was also used to detect loops of evidence for inconsistency[^5^](#_ENREF_5)^,^ [^6^](#_ENREF_6).

**Confidence in network meta-analysis**

Confidence in Network Meta-analysis (CINeMA) web application[^7^](#_ENREF_7) for the primary outcome i.e. final stone free rate. CINeMA requires data formatted in terms of study level outcome, risk of bias and indirectness. Data was then configured and network plot was created. Nodes were colored green, yellow or red according to risk of bias (low, unclear and high respectively). A bar graph depicting contributions of each study to network estimate is generated. For this given network estimate risk of bias across contributions was summarized by selecting “Average” command. For assessing imprecision a risk ratio of 1.25 was set as clinically important size of effect. Relative effect estimates below 0.8 and above 1.250 were considered clinically important. Judgment for imprecision were formulated as “very serious”, “serious” and “not serious” depending upon whether the confidence interval (CI) values cross both, one or neither limits of clinically important effect zones. Prediction intervals were generated to make judgments on heterogeneity and its implications on quality of treatment effects. Incoherence or inconsistency was assessed according to methods described on separate section for same. Finally, results of all comparisons were graded as high, moderate, low or very low according to this framework[^8^](#_ENREF_8).

Supplementary figure 1: Depicts network map, interval plot and Forest plot for need for auxiliary procedures.


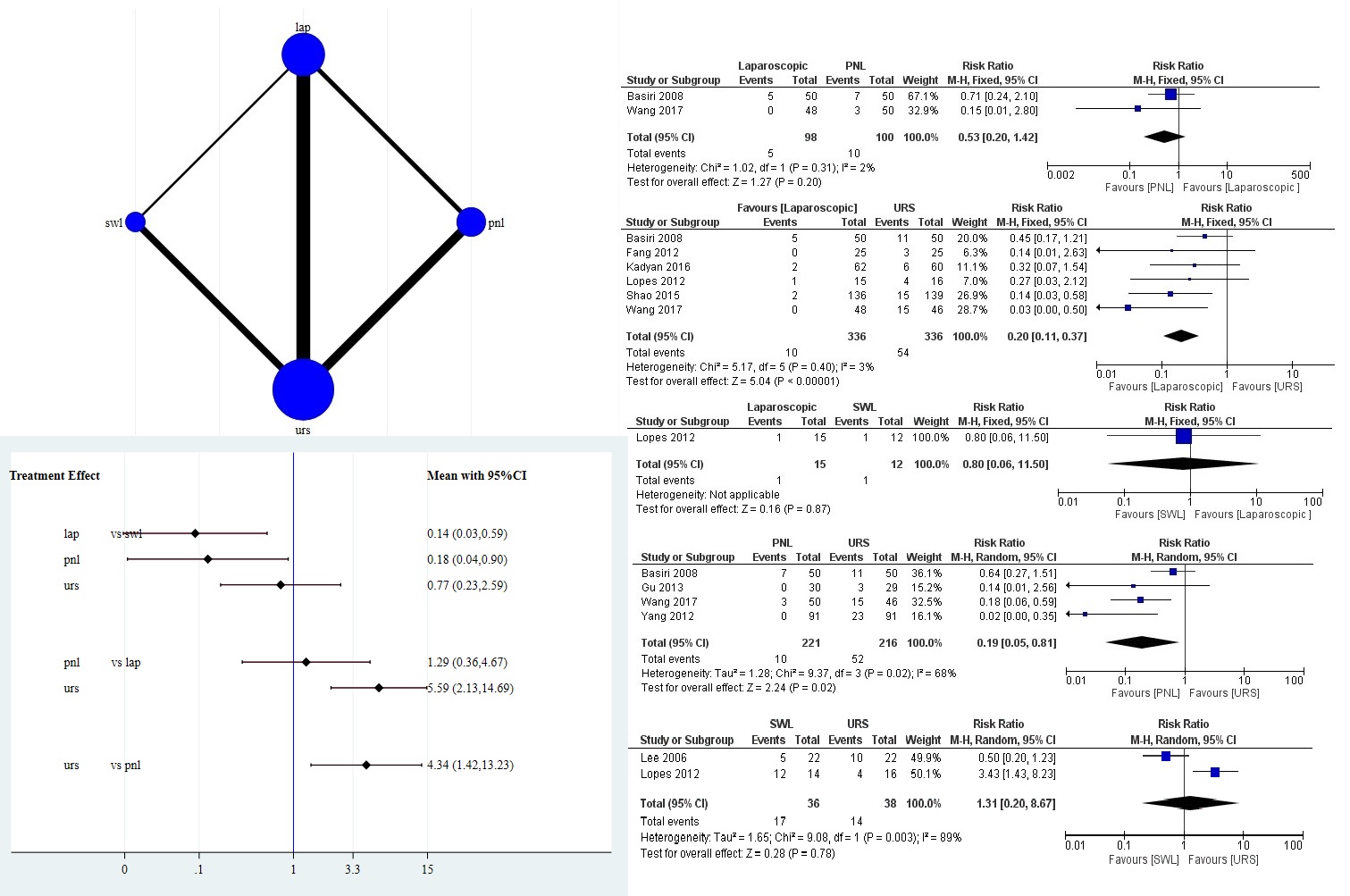


Supplementary figure 2: Depicts network map, interval plot and Forest plot for the duration of surgery.


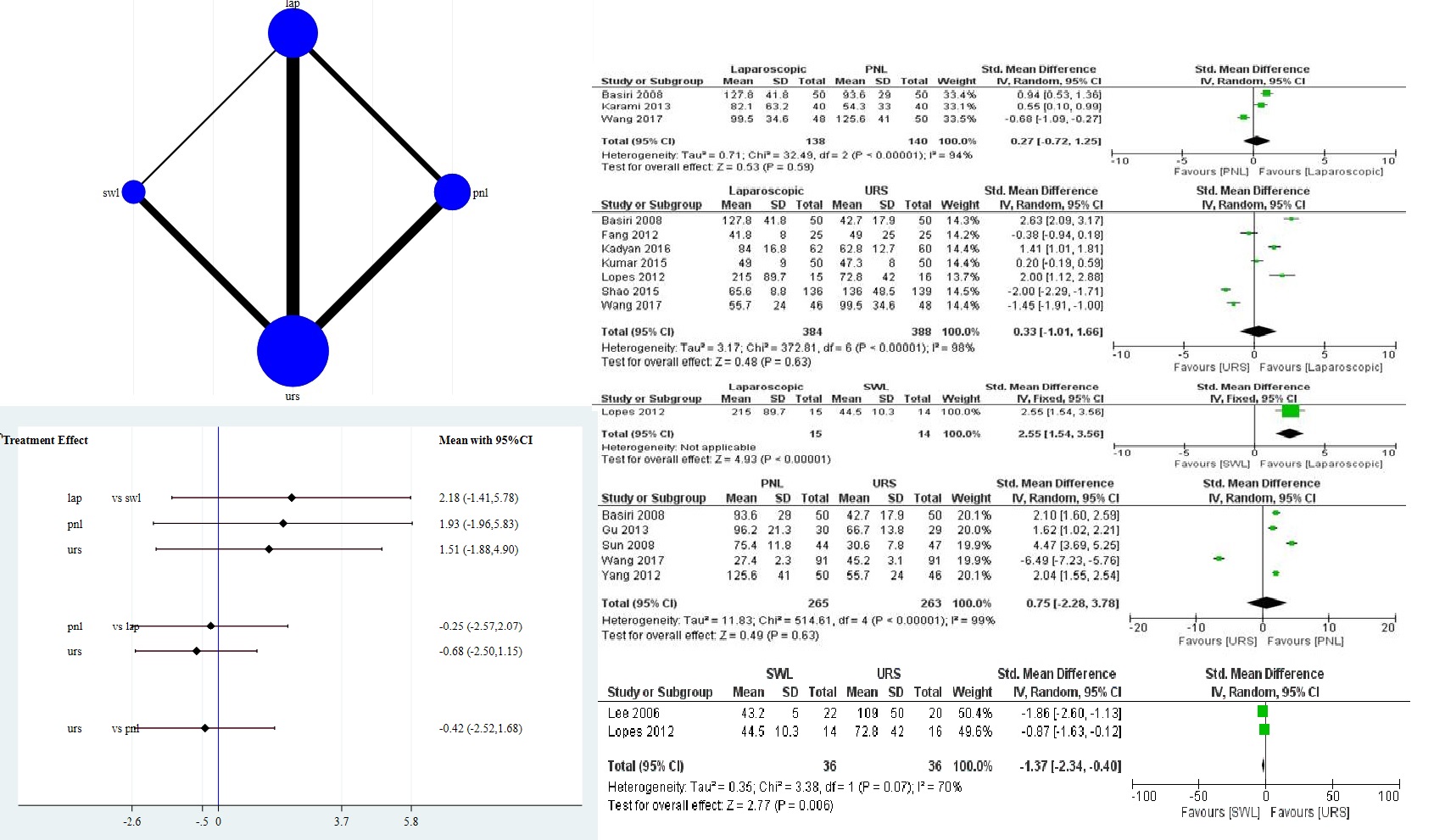


Supplementary figure 3: Depicts network map, interval plot and Forest plot for the length of hospital stay.


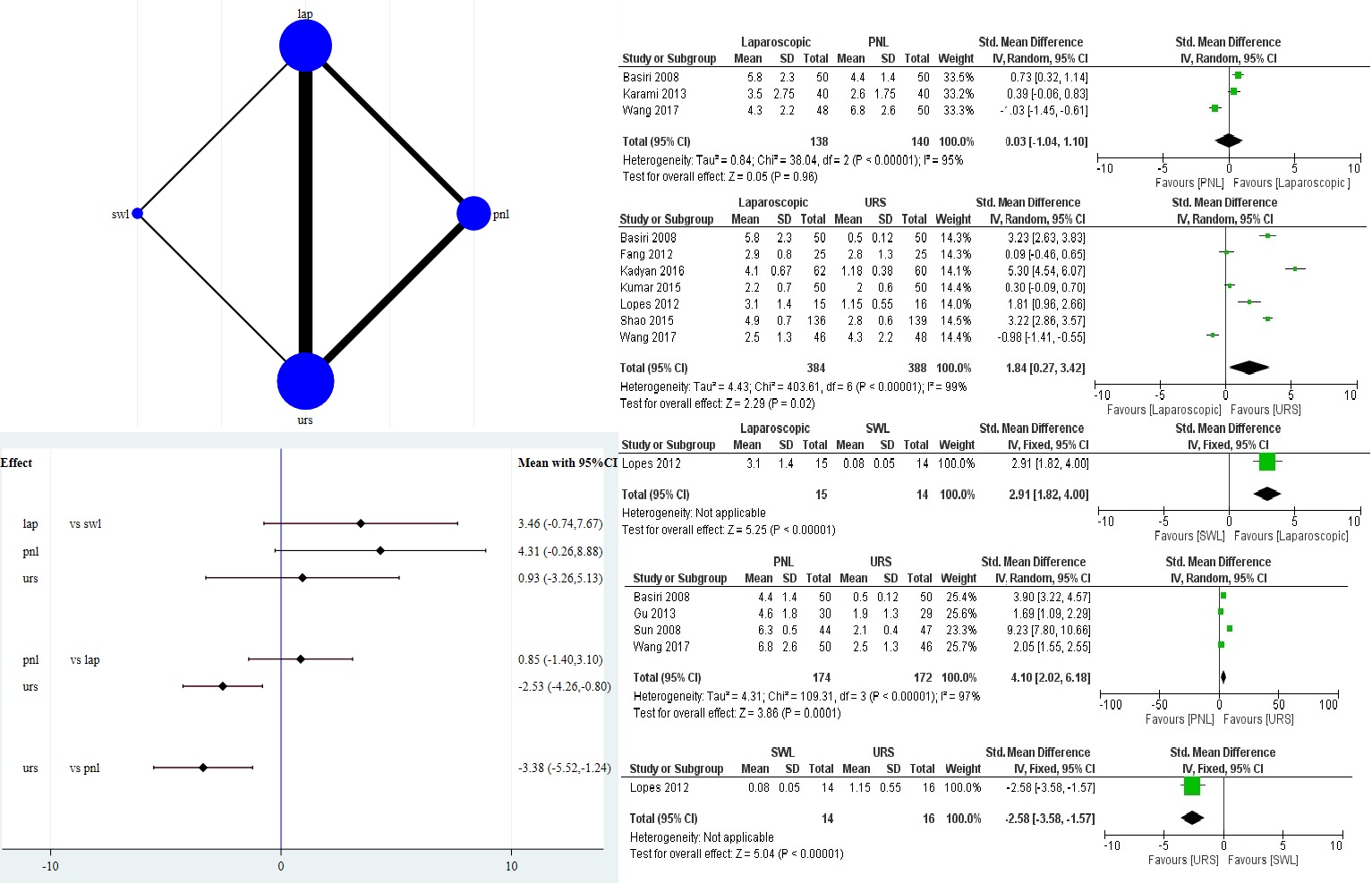


Supplementary figure 4: Depicts network map, interval plot and Forest plot for the overall complication rates.


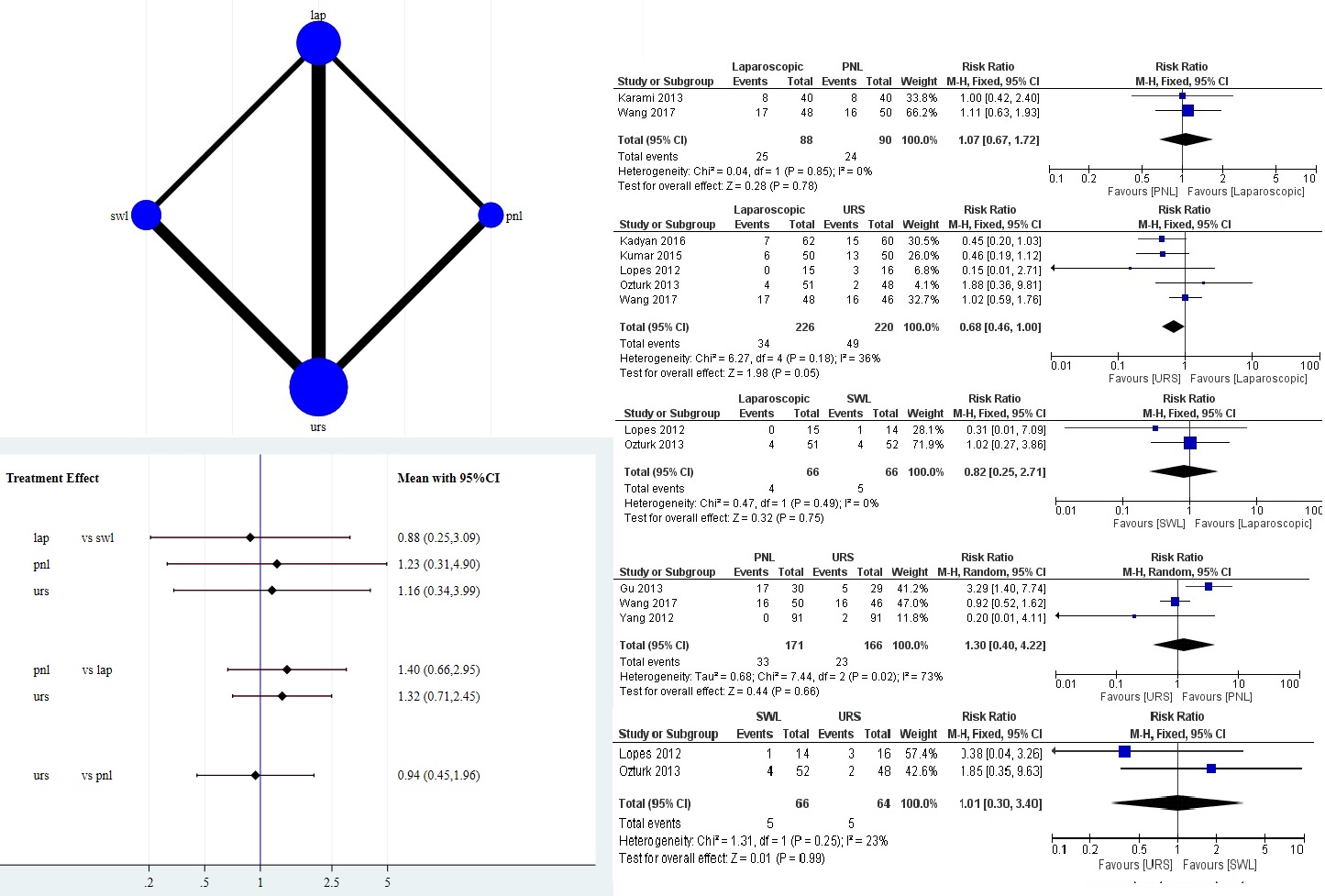


**Supplementary figure 5:** Loop specific approach to assess the inconsistency in various existing loops for treatment groups for various outcomes.


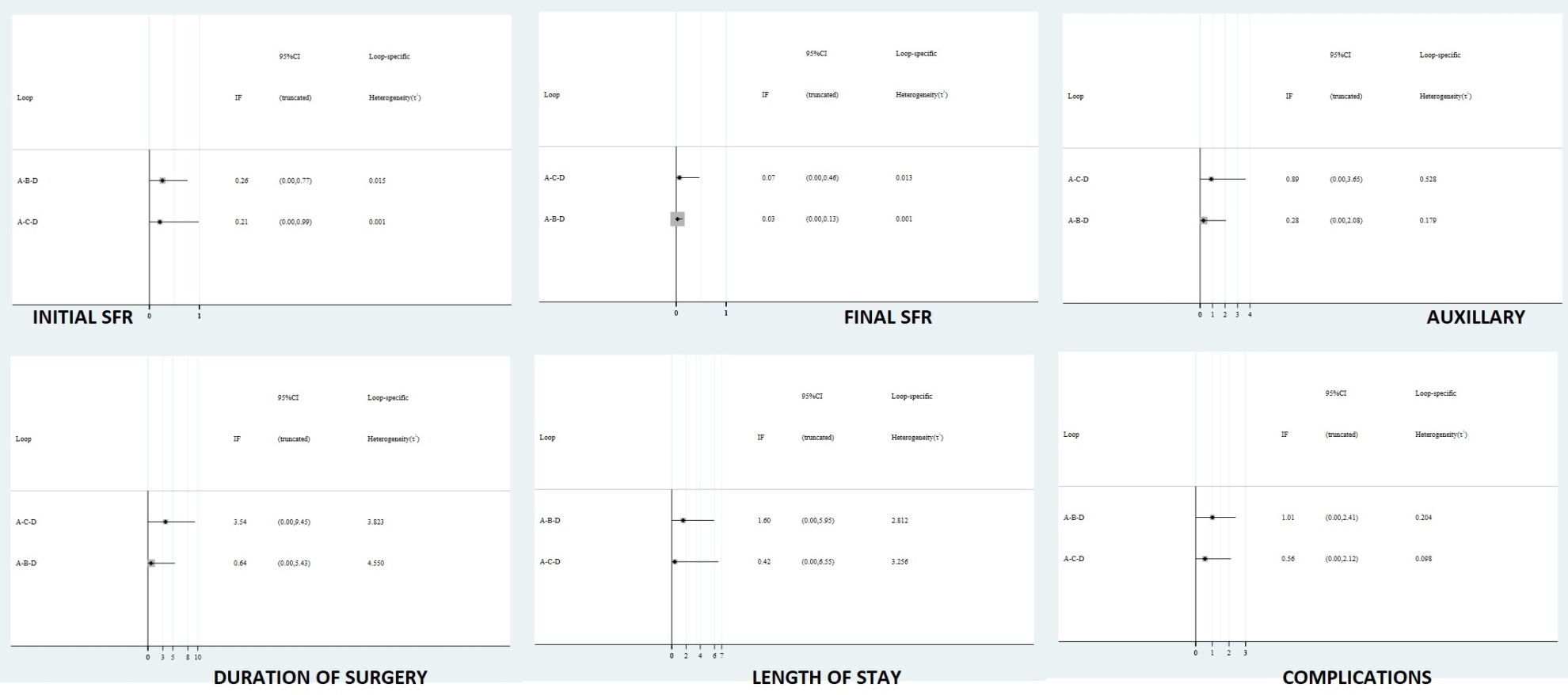


**Risk of bias and certainty of evidence**

Of the 13 studies included in the meta-analysis, 10 studies had adequately described randomization technique and were at low risk of bias for same. Allocation bias was adequately addressed only in three studies whereas rest of the studies had not described it and was at unclear risk of bias. No included studies had addressed the performance bias and were at unclear risk for same. Detection bias was addressed only by Ozturk et al[^9^](#_ENREF_9) where radiologist was blinded and rest of the studies were at unclear risk of bias. Attrition bias was absent in all the studies except for Lee et al[^10^](#_ENREF_10) and reporting bias was noted in three studies due to inadequate addressal of the outcomes.

**Supplementary Table 2:** Confidence for various comparing using CINeMA approach for primary outcome i.e. final SFR.

| **Comparison** | **Number of studies** | **Within-study bias** | **Reporting bias** | **Indirectness** | **Imprecision** | **Heterogeneity** | **Incoherence** | **Confidence rating** |
| --- | --- | --- | --- | --- | --- | --- | --- | --- |
| **lap:pnl** | 3 | Some concerns | Undetected | No concerns | Major concerns | No concerns | No concerns | Very Low |
| **lap:swl** | 2 | Some concerns | Undetected | Some concerns | No concerns | No concerns | No concerns | Low |
| **lap:urs** | 7 | Some concerns | Undetected | Major concerns | No concerns | No concerns | No concerns | Very Low |
| **pnl:urs** | 5 | Some concerns | Undetected | No concerns | No concerns | No concerns | No concerns | Moderate |
| **swl:urs** | 2 | Some concerns | Undetected | No concerns | Major concerns | No concerns | No concerns | Very Low |
| **pnl:swl** | 0 | Some concerns | Undetected | No concerns | No concerns | No concerns | No concerns | Moderate |

Supplementary Figure 6: Funnel plot for assessment of Publication bias for the primary outcome i.e. final stone free rate


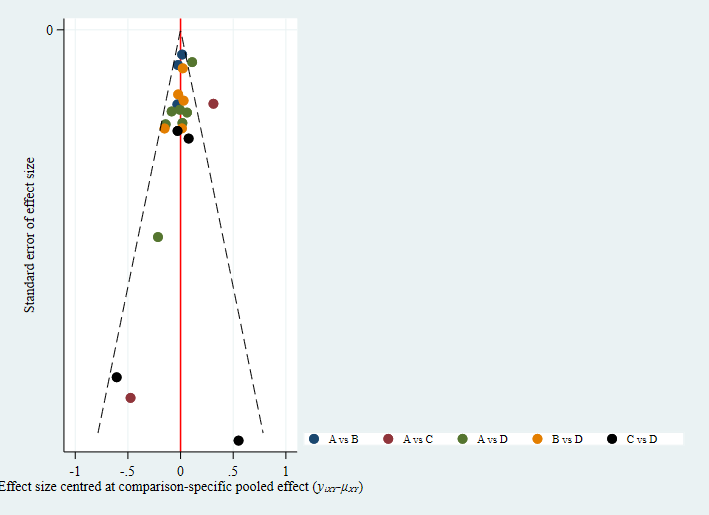


A= SWL; B= LUL; C=PNL; D= URS

**REFERENCES**

1. Hozo SP, Djulbegovic B, Hozo I. Estimating the mean and variance from the median, range, and the size of a sample. BMC Med Res Methodol. 2005;5:13.

2. StataCorp. Stata statistical software: release 16. College station, TX: StataCorp LLC; 2019.

3. White I. Network meta-analysis. Stata Journal. 2015;15:951-85.

4. Chaimani A, Higgins JP, Mavridis D, Spyridonos P, Salanti G. Graphical tools for network meta-analysis in STATA. PLoS One. 2013;8:e76654.

5. Higgins JP, Jackson D, Barrett JK, Lu G, Ades AE, White IR. Consistency and inconsistency in network meta-analysis: concepts and models for multi-arm studies. Res Synth Methods. 2012;3:98-110.

6. White IR, Barrett JK, Jackson D, Higgins JP. Consistency and inconsistency in network meta-analysis: model estimation using multivariate meta-regression. Res Synth Methods. 2012;3:111-25.

7. CINeMA: Confidence in network Meta-analysis [Software] Institute of Social and Preventive Medicine: University of Bern; 2017. Available from: cinema.ispm.ch.

8. Nikolakopoulou A, Higgins JPT, Papakonstantinou T, Chaimani A, Del Giovane C, Egger M, et al. CINeMA: An approach for assessing confidence in the results of a network meta-analysis. PLoS Med. 2020;17:e1003082.

9. Ozturk U, Can Şener N, Goksel Goktug HN, Gucuk A, Nalbant I, Abdurrahim Imamoglu M. The comparison of laparoscopy, shock wave lithotripsy and retrograde intrarenal surgery for large proximal ureteral stones. Journal of the Canadian Urological Association. 2013;7:E673-E6. English.

10. Lee YH, Tsai JY, Jiaan BP, Wu T, Yu CC. Prospective randomized trial comparing shock wave lithotripsy and ureteroscopic lithotripsy for management of large upper third ureteral stones. Urology. 2006;67:480‐4; discussion 4.
